# Development of a novel dynamic nomogram for predicting overall survival in anaplastic thyroid cancer patients with distant metastasis: a population-based study based on the SEER database

**DOI:** 10.3389/fendo.2024.1375176

**Published:** 2024-07-04

**Authors:** Liuhuan Wang, Yanghua Rao, Pengxing Lai, Yunxia Lv

**Affiliations:** The Second Affiliated Hospital, Jiangxi Medical College, Nanchang University, Nanchang, China

**Keywords:** anaplastic thyroid cancer, distant metastasis, SEER, overall survival, nomogram

## Abstract

**Background:**

Anaplastic thyroid cancer (ATC) is highly invasive, prone to distant metastasis (DM), and has a very poor prognosis. This study aims to construct an accurate survival prediction model for ATC patients with DM, providing reference for comprehensive assessment and treatment planning.

**Methods:**

We extracted data of ATC patients with DM diagnosed between 2004 and 2019 from the SEER database, randomly dividing them into a training set and a validation set in a ratio of 7:3. Univariate and multivariate Cox regression analyses were sequentially performed on the training set to identify independent prognostic factors for overall survival (OS) and construct nomograms for 3-month, 6-month, and 8-month OS for ATC patients with DM based on all identified independent prognostic factors. Receiver operating characteristic (ROC) curve analysis, decision curve analysis (DCA) curve analysis, and calibration curves were separately plotted on the training and validation sets to demonstrate the model’s performance. Furthermore, patients were stratified into high- and low-risk groups based on their risk scores, and the Kaplan-Meier (KM) survival curves were used to illustrate the survival differences between the two groups.

**Results:**

A total of 322 patients were included in this study. Univariate and multivariate Cox regression analyses identified five independent prognostic factors for OS in ATC patients with DM: surgery, tumor size, age, chemotherapy, and radiotherapy. Nomograms for 3-month, 6-month, and 8-month OS were established based on these factors. The training set AUC values (3-month AUC: 0.767, 6-month AUC: 0.789, 8-month AUC: 0.795) and validation set AUC values (3-month AUC: 0.753, 6-month AUC: 0.798, 8-month AUC: 0.806) as well as the calibration curves demonstrated excellent applicability and accuracy of the model. Additionally, the DCA curves indicated substantial clinical net benefit of the model. The KM curves also confirmed the model’s excellent stratification ability for patient OS.

**Conclusion:**

The nomogram developed in this study accurately predicts OS for ATC patients with DM. It can assist clinicians in formulating appropriate treatment strategies for these patients.

## 1 Introduction

Anaplastic thyroid cancer (ATC) is a rare but highly aggressive malignancy (1). Although it accounts for less than 2% of all types of thyroid cancer, it contributes to more than 50% of the annual mortality rate associated with thyroid cancer (2, 3). The median survival time for ATC is approximately four months (4), and the disease-specific mortality rate ranges from 98% to 99% (5, 6). The most notable clinical feature of ATC is its high invasiveness, which leads to local infiltration and metastasis to regional lymph nodes or distant organs (7). Around 50% of ATC patients are diagnosed with distant metastasis (DM) at the time of diagnosis (8, 9), resulting in a significantly worse prognosis. Current treatment options for ATC patients with DM mainly include surgery, radiotherapy, and chemotherapy (10). The selection of precise treatment strategies for individual patients relies on their comprehensive systemic evaluation and survival prediction. Given the highly malignant nature and rapid disease progression of this condition, it is crucial to develop appropriate assessment tools for ATC patients with DM to facilitate accurate management planning.

Although previous studies have contributed some practical clinical prediction tools (11–13), these tools encompassed all ATC patients. However, patients with ATC and DM often exhibit distinct clinical characteristics and biological behaviors compared to the overall population, representing a significant proportion of all ATC patients. Therefore, there is an urgent need to establish a precise clinical prediction tool specifically tailored to ATC patients with DM.

In this study, we included multicenter patient data from the Surveillance, Epidemiology, and End Results (SEER) database to evaluate independent prognostic factors influencing overall survival (OS) in ATC patients with DM. We constructed a nomogram to predict OS for this patient population and confirmed the excellent performance of the model through a series of evaluation metrics.

## 2 Materials and methods

### 2.1 Data source

The patient cohort for this study was derived from ATC patients with DM diagnosed between 2004 and 2019 in the SEER database. The clinical information of all patients was extracted from the SEER Cancer Database (http://www.seer.cancer.gov) using SEER*Stat software (https://seer.cancer.gov/seerstat/, version 8.4.2). As patient data in the SEER database is de-identified, local ethical review was not required for this study.

### 2.2 Patient selection criteria

The inclusion criteria for the patient cohort in this study were as follows: (1) primary tumor site in the thyroid; (2) histological diagnosis codes according to the International Classification of Diseases for Oncology, 3rd edition (ICD-O-3) as 8020/3 and 8021/3; (3) presence of distant metastasis, classified as stage IVC according to the eighth edition of the AJCC. Patients meeting any of the following criteria were excluded from this study: (1) cases provided by autopsy or death reports; (2) survival time of 0; (3) missing clinical information. All radiotherapeutic modalities involved in this study were external beam radiation therapy.

### 2.3 Statistical analysis

Considering the extremely high disease-specific mortality rate in the study population and the potential side effects of treatments such as radiation and chemotherapy, OS was chosen as the study endpoint. Firstly, all patients were randomly divided into training and validation sets in a 7:3 ratio, and the Chi-square/Wilcoxon test confirmed no statistical differences in baseline clinical characteristics between the two sets. Subsequently, univariate Cox regression analysis and multivariate Cox regression analysis were performed in the training set to determine the independent prognostic factors for OS in ATC patients with DM. A nomogram was constructed based on the selected independent risk factors. To evaluate the accuracy and clinical utility of the nomogram, receiver operating characteristic (ROC) curves, decision curve analysis (DCA) curves, and calibration curves were simultaneously plotted in both the training and validation sets. The ROC curve was used to assess the accuracy and recall of the model; the DCA curve was used to evaluate the clinical net benefit; the calibration curve was used to assess the prediction accuracy and consistency of the model. Risk scores for each patient were calculated based on the model, and patients were stratified into high- and low-risk groups using the median risk score. The Kaplan-Meier (KM) survival curves were employed to demonstrate the model’s ability to stratify patient prognosis. Finally, a web-based dynamic nomogram was published for readers’ use. All statistical analyses and visualizations in this study were performed using R software (version 4.3.1). A two-sided P value <0.05 was considered statistically significant.

## 3 Results

### 3.1 Patient characteristics

**Figure 1:**
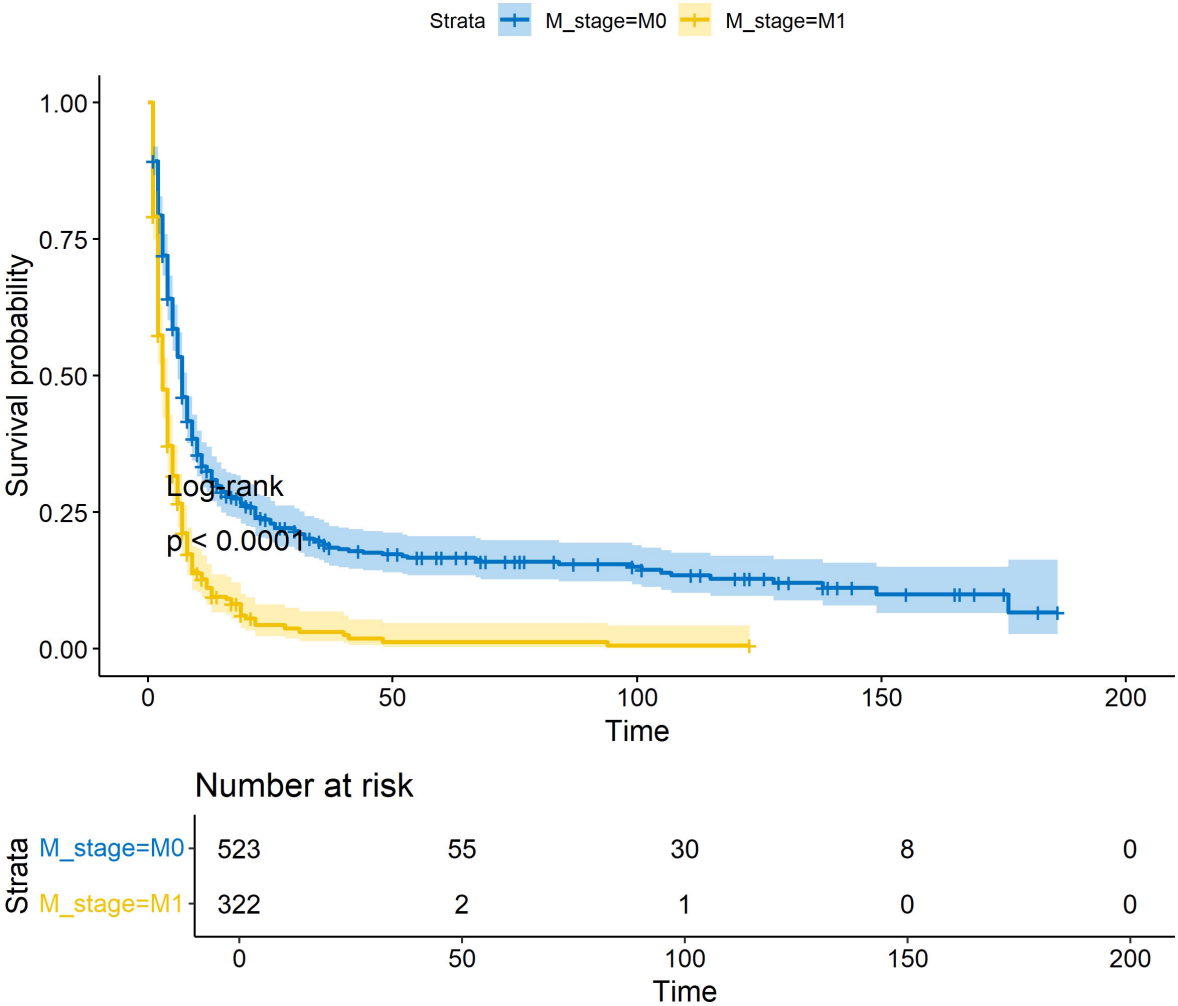
KM survival curves for OS in ATC patients with/without distant metastases. * p<0.05; ** p<0.01; *** p<0.001.

This study assimilated 322 qualified patients afflicted with ATC, with a median survival duration of three months. The Kaplan-Meier survival plot pertaining to the patient cohort in this investigation, juxtaposed with ATC sufferers devoid of distant metastasis, is demonstrated in **Figure 1** (**Figure 1**). As shown in **Table 1**, all patients in the study cohort had advanced T stage (T3a and above), with the majority of patients classified as T4b (72.4%). Similarly, the majority of patients had lymph node metastasis, with N1b being the most common stage (56.8%). Most patients received surgical treatment (48.1%), chemotherapy (51.2%), and radiotherapy (63.4%). The mean age for all patients stands at 68.2 (± 12.1). All patients were randomly allocated to the training and validation sets in a 7:3 ratio, and there were no statistically significant differences in clinical variables between the two groups (p>0.05, **Table 1**).

**Table 1:** Demographic and clinicopathological characteristics of ATC patients with distant metastases.

	Overall	Training cohort	Validation cohort	p-value
	(N=322)	(N=225)	(N=97)	
**Age**				0.727
Mean (SD)	68.3 (12.1)	68.2 (11.9)	68.4 (12.7)	
Median [Min, Max]	69.0 [23.0, 92.0]	69.0 [35.0, 92.0]	69.0 [23.0, 89.0]	
**Sex**				0.828
Female	168 (52.2%)	116 (51.6%)	52 (53.6%)	
Male	154 (47.8%)	109 (48.4%)	45 (46.4%)	
**Race**				0.586
White	255 (79.2%)	179 (79.6%)	76 (78.4%)	
Black	23 (7.1%)	14 (6.2%)	9 (9.3%)	
Other	44 (13.7%)	32 (14.2%)	12 (12.4%)	
**T stage**				0.288
T3a	11 (3.4%)	8 (3.6%)	3 (3.1%)	
T3b	12 (3.7%)	7 (3.1%)	5 (5.2%)	
T4a	66 (20.5%)	52 (23.1%)	14 (14.4%)	
T4b	233 (72.4%)	158 (70.2%)	75 (77.3%)	
**N stage**				0.744
N0	99 (30.7%)	69 (30.7%)	30 (30.9%)	
N1a	40 (12.4%)	30 (13.3%)	10 (10.3%)	
N1b	183 (56.8%)	126 (56.0%)	57 (58.8%)	
**Surgery**				0.916
No	167 (51.9%)	115 (51.1%)	52 (53.6%)	
Lobectomy/Isthmectomy	51 (15.8%)	36 (16.0%)	15 (15.5%)	
Subtotal/total thyroidectomy	104 (32.3%)	74 (32.9%)	30 (30.9%)	
**Tumor size**				0.960
Mean (SD)	64.1 (27.8)	63.8 (26.6)	64.7 (30.6)	
Median [Min, Max]	64.5 [2.00, 165]	64.0 [2.00, 152]	65.0 [3.00, 165]	
**Chemotherapy**				0.770
No	157 (48.8%)	108 (48.0%)	49 (50.5%)	
Yes	165 (51.2%)	117 (52.0%)	48 (49.5%)	
**Radiotherapy**				0.810
No	118 (36.6%)	81 (36.0%)	37 (38.1%)	
Yes	204 (63.4%)	144 (64.0%)	60 (61.9%)	

### 3.2 Prognostic factors for ATC patients with DM

To explore potential clinical prognostic factors associated with OS in ATC patients with DM, we performed univariate Cox regression analysis on nine potential factors, revealing five variables significantly associated with OS: age, tumor size, surgery, chemotherapy, and radiotherapy. Further multivariate Cox regression analysis (**Table 2**) demonstrated that age, tumor size, surgery, chemotherapy, and radiotherapy were independent prognostic factors for OS in ATC patients with DM. Specifically, chemotherapy (OR=0.622, 95%CI=0.469-0.826), and radiotherapy (OR=0.739, 95%CI=0.550-0.994) were protective factors for patient OS. However, T stage and N stage were not independent prognostic factors for OS (P>0.05).

**Table 2:** Univariate and multivariate Cox analyses in ATC patients with distant metastases.

	Univariate analysis			Multivariate analysis		
	OR	95%CI	P	OR	95%CI	P
**Age**	1.019	1.007-1.031	0.001^*^	1.013	1.001-1.025	0.031^*^
Sex						
Female	Reference					
Male	1.133	0.858-1.496	0.381			
Race						
White	Reference					
Black	1.028	0.582-1.816	0.924			
Other	1.131	0.766-1.671	0.535			
T stage						
T3a	Reference					
T3b	0.347	0.101-1.185	0.091			
T4a	0.660	0.297-1.466	0.307			
T4b	0.885	0.415-1.891	0.753			
N stage						
N0	Reference					
N1a	0.705	0.439-1.132	0.148			
N1b	1.334	0.983-1.810	0.064			
Surgery						
No	Reference					
Lobectomy/Isthmectomy excision	0.567	0.382-0.841	0.005^*^	0.664	0.445-0.993	0.046^*^
Subtotal/total thyroidectomy	0.517	0.375-0.712	<0.001^*^	0.624	0.448-0.869	0.005^*^
Chemotherapy						
No	Reference					
Yes	0.557	0.422-0.735	<0.001^*^	0.622	0.469-0.826	0.001^*^
Radiotherapy						
No	Reference					
Yes	0.619	0.465-0.823	<0.001^*^	0.739	0.550-0.994	0.045^*^
**Tumor size, mm**	1.012	1.007-1.018	<0.001^*^	1.011	1.006-1.017	<0.001^*^

* p<0.05, statistically significant.

### 3.3 Prognostic nomogram development and validation

**Figure 2:**
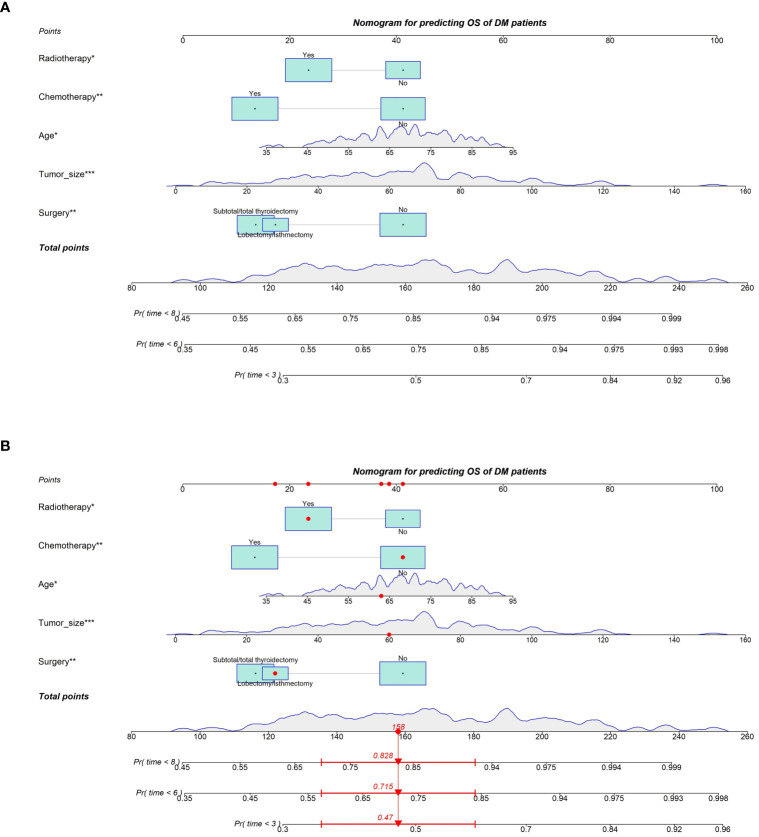
Prognostic nomogram predicting OS at 3, 6 and 8 months in SIC ATC patients with distant metastases **(A)**. Schematic of patient application model for prediction **(B)**.

**Figure 3:**
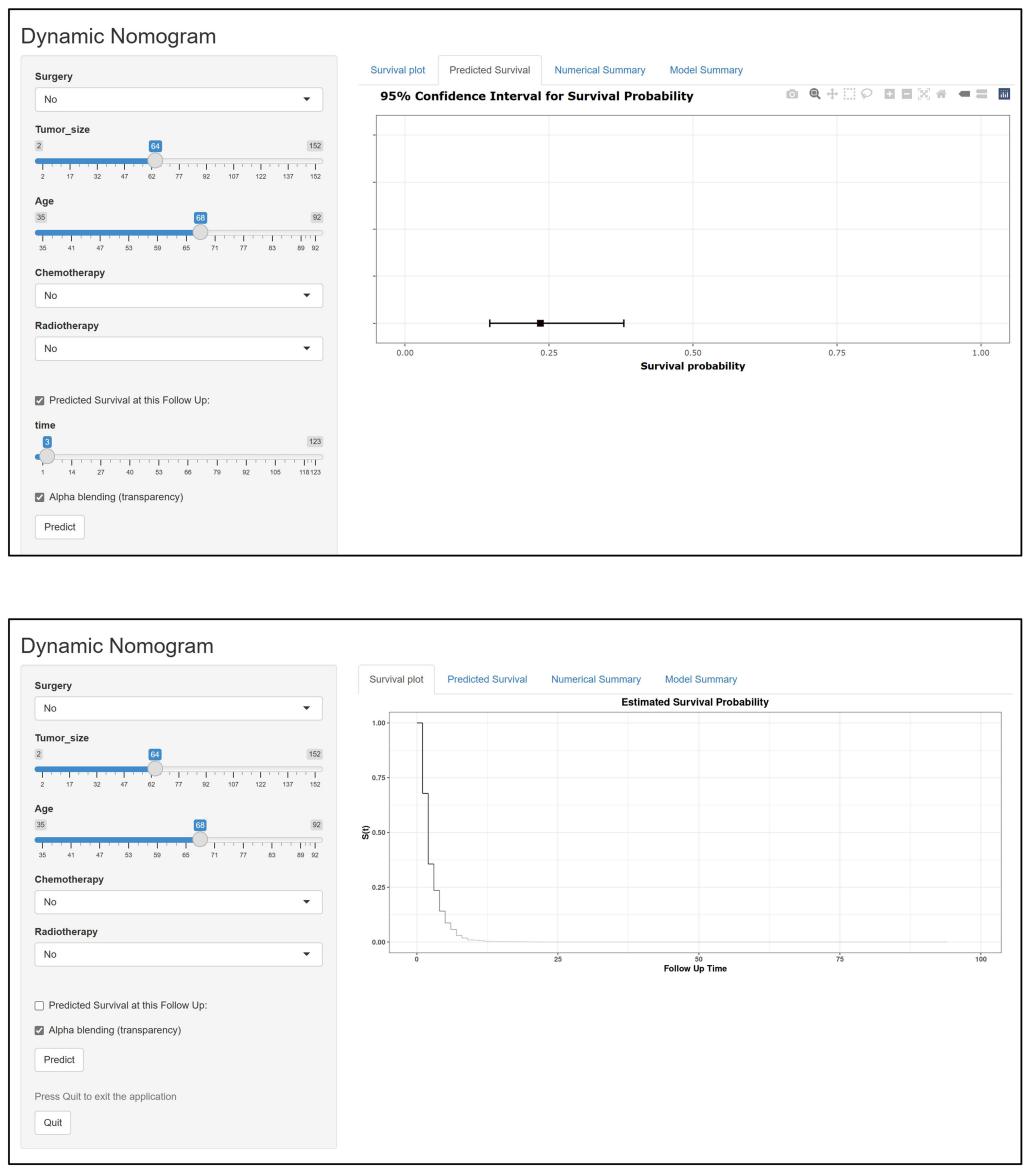
Schematic diagram of the interface for using the web version of the dynamic prediction model.

Based on the Cox regression analysis results of the five independent prognostic factors, we constructed a nomogram for predicting 3-month, 6-month, and 8-month OS in ATC patients with DM (**Figure 2A**). Each predictive variable was assigned a specific score based on its position on the corresponding scale, and the cumulative sum of all “points” yielded the “total points,” which could be further converted into the probability of death at a specific time point for a particular patient. We also provided a demonstration of survival prediction using randomly selected patient data (**Figure 2B**). Additionally, we published an online dynamic nomogram (https://jzxwlh.shinyapps.io/DynNomapp/) (**Figure 3**).

**Figure 4:**
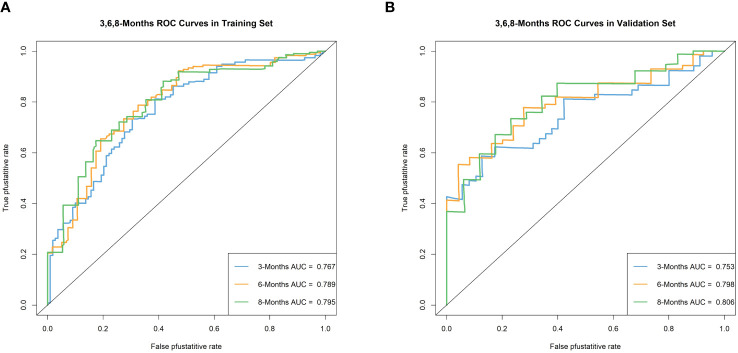
Time-dependent ROC curve analysis of the OS nomogram for the 3-, 6-, and 8-month in the training set **(A)** and the validation set **(B)**.

**Figure 5:**
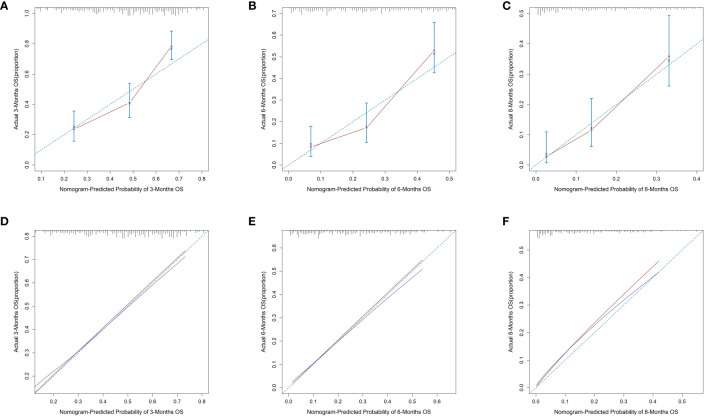
Calibration curves for 3-, 6-, and 8-month OS prediction nomogram in the training set **(A–C)** and validation set **(D–F)**.

**Figure 6:**
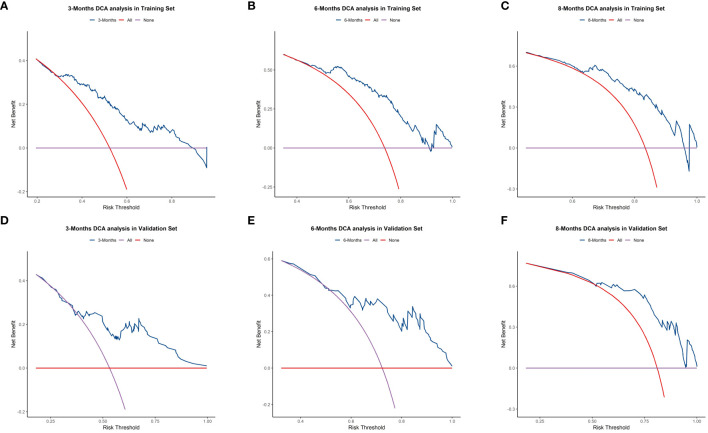
Decision Curve Analysis (DCA) curves for 3-, 6-, and 8-month OS prediction nomogram in the training set **(A–C)** and validation set **(D–F)**.

**Figure 7:**
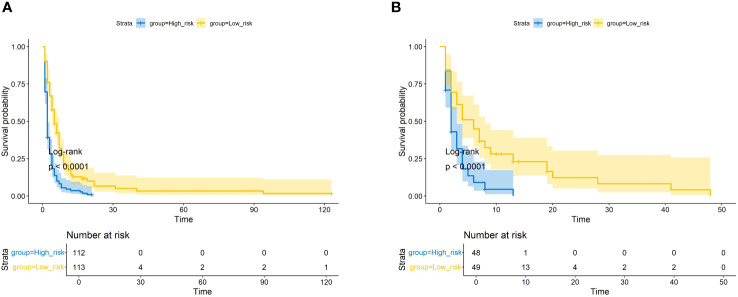
The Kaplan–Meier survival curves of the patients in the training set **(A)** and in the validation set **(B)**.

We plotted ROC curves in both the training and validation sets to evaluate the sensitivity and specificity of the model. The results showed that the model achieved AUC values of 0.767, 0.789, and 0.795 for 3-month, 6-month, and 8-month OS, respectively, in the training set (**Figure 4A**), and AUC values of 0.753, 0.798, and 0.806 for 3-month, 6-month, and 8-month OS, respectively, in the validation set (**Figures 4A, B**), confirming the excellent accuracy of the model. Calibration curves (**Figure 5**) and DCA curves (**Figure 6**) were plotted in both the training and validation sets. The calibration curves visually demonstrated the accurate performance of the nomogram in predicting OS at different time points, while the DCA curves confirmed the outstanding performance of the nomogram in real clinical practice. KM survival curves (**Figure 7**) illustrated the model’s excellent stratification ability for patient OS in both the training and validation sets.

## 4 Discussion

Despite the low incidence of ATC, its highly aggressive nature and poor prognosis have attracted increasing attention from scholars (14). Current mainstream treatment modalities for ATC patients include surgery, chemotherapy, radiotherapy, and other comprehensive treatments. However, the prognosis for ATC patients remains unfavorable (15). Therefore, it is crucial to actively study and explore prognostic factors in ATC patients to assess their risk. In this study, we focused on ATC patients with DM and utilized multicenter data from the SEER database to thoroughly investigate the independent prognostic factors in ATC patients with DM. We also developed a nomogram to assist in determining optimal treatment strategies for these patients. To the best of our knowledge, this study is the first multicenter retrospective study to construct a prognostic model specifically for ATC patients with DM. We further confirmed the excellent performance of the model through a series of evaluation metrics.

Thyroid cancer is the only cancer that considers age as an important prognostic factor for thyroid cancer-specific survival. Similarly, in our study, we observed a similar phenomenon where patients over 75 years of age had significantly worse prognosis compared to other age groups. Our constructed nomogram vividly illustrates this point, where older patients receive higher risk scores. Additionally, we found that larger tumor diameter was directly associated with poorer prognosis. Previous studies have highlighted the importance of tumor diameter differences in determining invasiveness (13), but specific research on the relationship between tumor diameter, distant metastasis, and prognosis in ATC is still lacking.

In our study cohort, we were surprised to discover that unlike most tumors, the extent of primary tumor infiltration and regional lymph node metastasis were no longer independent prognostic factors for ATC patients with DM. Instead, surgery, chemotherapy, and radiotherapy were identified as independent prognostic factors. Similar findings have been reported in previous studies (13). We speculate that this is due to the highly invasive nature of ATC, resulting in the majority of ATC patients with DM being diagnosed at an advanced stage where the status of the primary tumor no longer significantly influences patient prognosis.

Furthermore, we noted that various treatment modalities were independent prognostic factors for OS in ATC patients with DM. This emphasizes the importance of actively receiving treatment after diagnosis to prolong patient survival. Previous research has shown that although surgery is indeed a protective factor for patient OS, it is not sufficient to improve long-term prognosis significantly, leading to ongoing debates regarding the extent of the benefits of surgical treatment (16, 17). The guidelines of the American Thyroid Association (18) explicitly state that the surgical goal for patients with ATC at stages IVA/IVB is to perform a resection of all macroscopically visible tumor (R0 or R1) rather than debulking (R2 resection). Additionally, they mention that a subset of patients at stage IVC may consider surgery to control local disease and alleviate or prevent future complications (for instance, impending airway invasion/obstruction, esophageal invasion/obstruction, laryngeal invasion/obstruction), albeit without elaborating in detail on specific surgical recommendations. In this study, we observed superior overall survival (OS) in patients with stage IVC ATC who underwent total/subtotal thyroidectomy compared to those who had only lobectomy/isthmectomy or didn’t undergo surgery. Thus, based on our analysis, we posit that debulking surgery targeting the primary tumor can enhance patients’ OS to some extent. Concurrently, guidelines from the American Thyroid Association also indicate that radical surgeries (including laryngectomy, tracheotomy, esophagectomy and/or major vascular or mediastinal resection) are generally not recommended due to the poor prognosis of ATC. Such procedures can be selectively contemplated only after comprehensive discussions within a multidisciplinary team, considering factors including patient mutation targets.

In our study, radiotherapy was found to be the most commonly used treatment modality for ATC patients with DM. According to previous literature, a radiation dose of at least 50 Gy is required to significantly improve patient prognosis (19, 20), and Pezzi et al (21) found that patients receiving radiation doses of 60-75 Gy had better survival rate improvements compared to those receiving lower doses. The NCCN guidelines recommend an adjuvant radiation dose of 60-66 Gy (14). Due to the low incidence and high invasiveness of ATC, patient enrollment in clinical trials evaluating various chemotherapy agents is extremely limited, leading to incomplete evaluation of chemotherapy efficacy in ATC (22). Regarding comprehensive treatments, many previous studies have consistently suggested that surgical treatment combined with adjuvant chemoradiotherapy can effectively improve OS in ATC patients (23, 24), aligning with our study results. However, some studies have found that while concurrent chemoradiotherapy and/or chemotherapy can improve survival rates in stage IVA/B ATC patients, the benefit is limited in stage IVC patients (25). Nevertheless, our study found that stage IVC patients who actively pursued various treatment options had significantly better prognoses than those who did not receive any treatment. Therefore, despite generally poorer prognoses for stage IVC ATC patients, we still advocate for their active treatment. Additionally, there is a pressing need for new treatment strategies to improve the prognosis of late-stage ATC patients.

In recent years, targeted therapy and immunotherapy for ATC have garnered increasing attention. Currently, BRAF mutation is identified as the most common somatic mutation in ATC. The drugs Dabrafenib and Trametinib, targeting BRAF and MEK1/2 respectively, work by inhibiting tumor cell proliferation through disruption of the RAF-MEK-ERK signaling pathway. Clinical studies suggest that the confirmed overall response rate to combined Dabrafenib and Trametinib therapy stands at 69%, with independently reviewed results corroborating this finding. This validates the significant clinical efficacy of combined Dabrafenib and Trametinib therapy in patients with BRAF V600E-mutant ATC (26, 27). Immunotherapy for ATC is still in the experimental stage; however, immune checkpoint inhibitors targeting cytotoxic T-lymphocyte-associated antigen 4 and PD-1/PD-L1 have shown promising clinical efficacy (28, 29). Unfortunately, ATC patient data on targeted/immunotherapies are not recorded in the SEER database, limiting our ability to conduct detailed analyses or incorporate these treatment variables into predictive models.

Of course, we must acknowledge certain limitations of our study. Firstly, as a retrospective analysis, we excluded patients with missing clinical information, which may introduce some selection bias. Secondly, although we included multicenter patient data spanning over a decade, the limited incidence of ATC with DM resulted in an inadequate number of patients in our study. Furthermore, as the SEER database does not include information regarding chemotherapy regimens, radiation dosage, and targeted/immunotherapy treatments, our predictive model did not take these factors into consideration. Lastly, we lack external validation sets to further validate the model. We hope that future researchers can include a larger number of patient data to further enhance our current research findings.

## 5 Conclusion

In this study, we identified the independent prognostic factors for OS in ATC patients with DM through univariate and multivariate Cox regression analysis. We developed a nomogram based on these factors and also released an online version of the dynamic nomogram. Furthermore, we demonstrated the excellent performance of the model through a series of evaluation metrics.

## Data availability statement

Publicly available datasets were analyzed in this study. This data can be found here: https://seer.cancer.gov/.

## Author contributions

LW: Conceptualization, Software, Writing – original draft. YR: Methodology, Visualization, Writing – original draft. PL: Software, Visualization, Writing – original draft. YL: Data curation, Funding acquisition, Investigation, Software, Validation, Visualization, Writing – review & editing.
