# Supplementary material for: Development of a novel dynamic nomogram for predicting overall survival in anaplastic thyroid cancer patients with distant metastasis: a population-based study based on the SEER database
Source: Front Endocrinol (Lausanne). 2024 Jul 4;15:1375176. doi: 10.3389/fendo.2024.1375176 (PMC11254621; doi:10.3389/fendo.2024.1375176)
Supplement: Supplementary file 1 [file Table_1.docx]

|  | **Overall** |
| --- | --- |
|  | **(N=256)** |
| **Bone** |  |
| No | 202 (78.9%) |
| Yes | 54 (21.1%) |
| **Liver** |  |
| No | 228 (89.1%) |
| Yes | 28 (10.9%) |
| **Lung** |  |
| No | 52 (20.3%) |
| Yes | 204 (79.7%) |
| **Brain** |  |
| No | 244 (95.3%) |
| Yes | 12 (4.7%) |

sTable1: Summary of major distant metastatic sites in patients diagnosed with anaplastic thyroid cancer during IVC between 2010 and 2019

|  | **Univariate analysis** | | | **Multivariate analysis** | | |
| --- | --- | --- | --- | --- | --- | --- |
|  | **OR** | **95%CI** | **P** | **OR** | **95%CI** | **P** |
| **Bone metastasis** |  |  |  |  |  |  |
| No | Reference |  |  |  |  |  |
| Yes | 0.975 | 0.709-1.341 | 0.878 |  |  |  |
| **Liver metastasis** |  |  |  |  |  |  |
| No | Reference |  |  |  |  |  |
| Yes | 1.303 | 0.865-1.963 | 0.206 |  |  |  |
| **Lung metastasis** |  |  |  |  |  |  |
| No | Reference |  |  |  |  |  |
| Yes | 1.287 | 0.929-1.784 | 0.129 |  |  |  |
| **Brain metastasis** |  |  |  |  |  |  |
| No | Reference |  |  |  |  |  |
| Yes | 1.296 | 0.704-2.386 | 0.404 |  |  |  |

sTable2: Cox regression analysis of metastatic sites in patients with anaplastic thyroid cancer diagnosed during IVC between 2010 and 2019
